# Willingness-to-Pay for Community-Based Health Insurance among Informal Workers in Urban Bangladesh

**DOI:** 10.1371/journal.pone.0148211

**Published:** 2016-02-01

**Authors:** Sayem Ahmed, Mohammad Enamul Hoque, Abdur Razzaque Sarker, Marufa Sultana, Ziaul Islam, Rukhsana Gazi, Jahangir A. M. Khan

**Affiliations:** 1 Health Economics & Financing Research Group, Centre for Equity and Health Systems, International Centre for Diarrhoeal Disease Research, Dhaka, Bangladesh; 2 Health Economics and Policy Research Group, Department of Learning, Informatics, Management and Ethics (LIME), Karolinska Institutet, Stockholm, Sweden; 3 School of Population Health, University of Queensland, Brisbane, Australia; 4 James P Grant School of Public health, BRAC University and International Centre for Diarrhoeal Disease Research, Dhaka, Bangladesh; 5 Liverpool School of Tropical Medicine, Pembroke Place, Liverpool, United Kingdom; New York University, UNITED STATES

## Abstract

**Introduction:**

Reliance on out-of-pocket payment for healthcare may lead poor households to undertake catastrophic health expenditure, and risk-pooling mechanisms have been recommended to mitigate such burdens for households in Bangladesh. About 88% of the population of Bangladesh depends on work in the informal sector. We aimed to estimate willingness-to-pay (WTP) for CBHI and identify its determinants among three categories of urban informal workers rickshaw-pullers, shopkeepers and restaurant workers.

**Methods:**

The bidding game version of contingent valuation method was used to estimate weekly WTP. In three urban locations 557 workers were interviewed using a structured questionnaire during 2010 and 2011. Multiple-regression analysis was used to predict WTP by demographic and household characteristics, occupation, education level and past illness.

**Results:**

WTP for a CBHI scheme was expressed by 86.7% of informal workers. Weekly average WTP was 22.8 BDT [Bangladeshi Taka; 95% confidence interval (CI) 20.9–24.8] or 0.32 USD and varied significantly across occupational groups (p = 0.000) and locations (p = 0.003). WTP was highest among rickshaw-pullers (28.2 BDT or 0.40 USD; 95% CI: 24.7–31.7), followed by restaurant workers (20.4 BDT 0.29 USD; 95% CI: 17.0–23.8) and shopkeepers (19.2 BDT or 0.27 USD; 95% CI: 16.1–22.4). Multiple regression analysis identified monthly income, occupation, geographical location and educational level as the key determinants of WTP. WTP increased 0.196% with each 1% increase in monthly income, and was 26.9% lower among workers with up to a primary level of education versus those with higher than primary, but less than one year of education.

**Conclusion:**

Informal workers in urban areas thus are willing to pay for CBHI and socioeconomic differences explain the magnitude of WTP. The policy maker might think introducing community-based model including public-community partnership model for healthcare financing of informal workers. Decision making regarding the implementation of such schemes should consider worker location and occupation.

## Introduction

Reliance on out-of-pocket (OOP) payments for healthcare increases the financial burden of households and causes impoverishment [1–4]. OOP spending is the major payment strategy for healthcare in most low and middle income countries, including Bangladesh. In Bangladesh OOP payments comprise 63.3% of total healthcare expenditure [5,6]. In a study of 11 Asian countries including Bangladesh, the investigators reported that OOP payments for healthcare impoverish 5 million people annually in Bangladesh [7]. Another study found the people of Bangladesh to exhibit the highest incidence of catastrophic health expenditure (15.57%) among 14 Asian countries [8].

Bangladesh has made remarkable progress in expanding coverage for essential public health interventions, such as immunization, which has markedly reduced maternal and child mortality rates [9]. However, coverage for secondary and tertiary care health services remains very limited, especially to the poor and vulnerable segments of society [10]. The government of Bangladesh spent only 629.8 BDT [(Bangladeshi Taka) (6.2 USD)] per capita on healthcare during 2012,while per capita OOP expenditure on health totaled 1,723.0 BDT (17.1 USD)[5]. In Bangladesh, private health expenditure constitutes 68.6% of total healthcare expenditure, of which 92.3% is covered through OOP payments[5]. In this context, despite significant improvement in numerous health indicators, availability of resources for health remains inadequate and financial protection for health expenditures is limited. On average around 15.6% of households faced catastrophic health expenditure because of the high burden of OOP payments [8].The WHO has determined that OOP payments are the least effective way to pay for healthcare [11].

While tax revenue and micro health insurance are two possible mechanisms for financing healthcare for low income citizens in Bangladesh, the former is currently insufficient because the government allocates only a small portion of its budget to healthcare (just 4.2% of government budget in 2012–13) and even that minimal commitment is subject to political interference [12].The tax-base in Bangladesh is small and the inclusion of low-income people (especially informal workers) in the tax system remains challenging and may not sufficient for this large group of population. The self-financed health schemes can be new source of financing beside tax revenue.

The International Labour Office(ILO) defines informal workers as own-account workers (excluding administrative workers and professionals), unpaid family workers, and employers and employees working in establishments with less than 10 staff [13]. Considering the importance of informal workers to the economy of Bangladesh, where they comprise 88% of the labor force and contribute 64% of GDP [14], efforts to attract these people to self-financing for healthcare are important.

Risk-pooling mechanisms are recommended to mitigate the consequences of dependence on OOP healthcare payments and to finance healthcare and help achieve universal coverage. The common/known consequences are suffering severe financial difficulties and falling into poverty[1–4]. Moreover, due to the large unpredictable OOP payment for healthcare the household often force to choose harder coping mechanisms like, borrowing with interest, asset selling [15]. The inclusion of informal workers in mutual insurance includes such challenges as making contributions or premiums more affordable for the poorest; increasing the range of services offered and the proportion of total costs covered; and improving financial management[16].

Occupational associations can provide a platform via which to engage with such workers regarding healthcare financing[10,17]. The Healthcare Financing Strategy of Bangladesh proposed extending health coverage to the entire population together with mechanisms for financing this, and showed that 85.7 million people (56.2% of the population) or 19.0 million households are connected to the informal sector and could be targeted by tax-funded healthcare, community-based health insurance (CBHI), micro health insurance, social protection schemes and other innovative initiatives for healthcare financing[10]. Community based health financing or CBHI is predominantly used for collective action in raising, pooling, allocating, purchasing, and supervising health financing arrangements, and is designed to spread costs and risks among members [18]. Long term experience with such schemes remains limited.

In the absence of data on actual health insurance usage, economists gauge willingness-to-pay (WTP) for health insurance in low-income countries via contingent valuation methods (CVM) which directly elicit what individuals would be willing to pay for a hypothetical health insurance package[19]. However, few studies have sought to understand WTP for health insurance in Bangladesh. This study aims to estimate WTP for CBHI and identify its determinants among selected groups of urban informal workers.

## Materials and Methods

### Ethics Statement

Informed written consent was taken from all interviewees, and confidentiality and anonymity were ensured. This study was approved by the Institutional Review Board of the International Centre for Diarrhoeal Disease Research, Bangladesh (icddr,b).

### Setting and sample

Three informal occupational groups (rickshaw-puller, shopkeepers and restaurant workers) were selected as study participants in three locations in Dhaka (a metropolitan city), Chandpur (a district town) and Nobinagar/Savar (a sub-district). These occupational groups were selected for investigation based on their prevalence throughout all urban areas in Bangladesh. The locations were selected to represent three levels in the urban administrative hierarchy of Bangladesh and thus achieve a national urban representation.

A sampling frame comprising all informal workers in the selected study locations did not exist because informal workers are not officially registered. However, a number of formal or informal worker cooperatives exist in all areas. To identify the study participants, we identified worker cooperatives and market places using transect walks and informal group discussions with community members and leaders. A list of workers was collected from the representatives/leaders of cooperatives or market places. A number of inclusion and exclusion criteria were applied. The inclusion criteria were age (18 years or above) and experience (working in the same occupation for at least the past year). The exclusion criteria were workers having health insurance or health insurance education [20]. Finally, we randomly selected 594 participants from the list of potential subjects. Data were collected by trained interviewers during 15 December, 2010 to 15 April, 2011 in all study areas. Among the 594 selected participants, 557 responded to the survey.

### Data collection tool

The informal worker was interviewed through a structured questionnaire. The worker was asked for demographic characteristics, household characteristics, monthly income and expenditure, health seeking behavior and bidding questions on WTP for health insurance. The monthly income information was asked separately to the worker for himself and the other member of his household. The household income from other sources (like, renting agriculture land, savings and fixed asset) was also collected. However, household expenditure data was not collected separately for specific items (like, food, clothing, and utility) rather the workers were asked for monthly average expenditure.

### Willingness to pay measurement

CVM was used to measure WTP for health insurance. This method has previously been used in many studies[21–23]. CVM questions can be either open-ended or discrete[24]. In an open-ended valuation the respondents are asked to state their maximum WTP for the benefit, typically using the so called “bidding game”. A bidding game resembles an auction, where a first bid is made to a respondent who then either accepts or rejects. Depending on the answer, the bid is then adjusted until the respondent’s maximum WTP is reached. This bidding game approach is applied to estimate WTP for health insurance. The “bidding game” has recently been employed by several studies to estimate WTP for CBHI in low and middle income countries[19,22]. The bidding game may be accompanied by estimation bias, which is a form of framing effect where respondents’ answers are influenced by the first numbers presented in the bidding game[25]. However, some studies have used the bidding game without observing any starting point bias[26,27].To determine appropriate starting bids, we interviewed numerous workers from each occupational group and questioned them regarding appropriate prices for CBHI. Based on the interview results we set a range from 10 to 30 BDT, and random figures in this range were included in individual questionnaires as the starting bids.

The benefit package, which is the same as that offered by Gonoshasthaya Kendra against a pre-paid membership card, was tested to investigate the WTP of workers for hypothetical CBHI scheme. The product together with associated copayment is presented briefly in Table 1. There were no deductibles in this hypothetical benefit package. Along with the services presented in Table 1, the financing mechanisms, and the terms and conditions under the hypothetical CBHI were explained in details to the respondent before proceeding to ask the WTP. The interviewer presented to the worker that four members of his household will be covered through this package for one year period if the worker enrolled in this package. He further explained outpatient health service will be provided through own doctor and medical paramedics of the CBHI scheme and inpatient care will be provided through the contracted public and private hospitals available locally. It was specified that there will be no super specialty hospitals in the contracted hospital list. The insurance scheme will be managed through a government registered cooperative where a management committee will be formed consisting the enrollees. The ministry of Local Government and Rural Development will monitor the cooperative on regular basis.

**Table 1:** The service package of the health insurance product.

Health services	Co-payment
**Outpatient**	
*Medical officer visit*	Free of cost
*Specialist visit*	60 BDT
**Inpatient**	
*Bed-Payment per day*	50 BDT
**Diagnostic tests**	
Ultra-sonography	75–150 BDT
ECG	50 BDT
Most of the low cost tests (Like, Blood grouping, Hb%, Stool test, Random Blood Sugar)	Free of cost
Some tests (like, Blood TC/DC/ESR, Urine RE,)	10–200 BDT
Blood transfusion of neonatal	500 BDT
Other treatment of neonatal	Free of cost
Normal delivery	100–500 BDT
Caesarean and other surgery	2000–3000 BDT
Orthopedic surgery	3000–4000 BDT
Appendicitis	100 BDT
Gall bladder operation	3000 BDT
**Medicine**	50% discount on maximum retail price set by government

The medical terminologies in the benefit package were explained to the respondent in local language. The interviewers were trained to present the medical terminologies in locally understandable language. The Field Research Supervisor monitored the process of presenting benefit package to the respondent. He helped data collector if there was any confusion on the medical terminologies in the benefit package at field site.

After explaining the benefit package and health insurance mechanism, each respondent was asked if he or she was willing-to-enroll in the CBHI scheme with his or her family members. The bidding game was then employed to determine the maximum price (premium) that a respondent will be willing-to-pay for the hypothetical CBHI scheme coverage for the four-member of household. The interviewer asked the respondent if he/she was willing-to-pay the randomly set amount as a starting bid. If the worker agreed, the interviewer would raise the bid and again question their WTP. The interviewer would then continue until the worker expressed unwillingness-to-pay. Conversely, if the worker expressed unwillingness-to-pay the starting bid, the interviewer would lower the bid and repeat the query, continuing until they reached a figure (including zero) that the worker was willing to pay.

### Data analysis

#### Descriptive analysis

The total household income adjusted for household size using OECD equivalence scale. This scale assigns a value of 1 to the first household member, of 0.7 to each additional adult and of 0.5 to each child [28]. Then the adjusted size of household was used to divide total household income to get the per-equivalent adult income. Similar approach was adopted to estimate per equivalent adult expenditure. Monthly income was used to create five income quintiles which were used to observe the association between income and WTP for CBHI scheme.

Mean and median WTP were estimated directly from the collected data. One way ANOVA test was conducted to test the difference in mean WTP across different occupational groups and areas. In total 13 outliers among WTP responses were identified using the approach proposed by Hadi, 1994 for the detection of outliers [29]. The mean WTP after removing outliers were presented separately. We used STATA version 11 for the statistical analyses. The minimal dataset underlying the findings of this study has been provided as supporting information (see S1 Dataset).

#### Econometric model

In the regression model, we predicted natural logged WTP based on respondent demographic and socioeconomic characteristics. Folland et al. (2007) produced a theoretical model in which premium, income or wealth, health status and risk of income loss can affect demand for health insurance[30]. Other researchers have identified similar factors in the empirical investigations[31–34]. The model below is used in the analysis:

$$\begin{array}{l} \ln(Y_{i})=\beta_{0}+\beta_{1}X_{1i}+\beta_{2}X_{2i}+\ldots +\varepsilon_{i}\\ i=1,2,\ldots ,n \end{array}$$

where Y_i_ denotes natural logged WTP for joining an insurance scheme, β_0_ is a constant,X_1_, X_2_, X_3_,……, X_n_ denote the control variables,β_1_, β_2_, β_3_,……β_n_ represents the coefficient that shows the magnitude and direction of the relationship of corresponding variables with Y, and ε is an error term. Because we used the natural logarithm of WTP as the dependent variable, the coefficients represented either semi-elasticities (if the independent variable is in natural units, e.g. age) or elasticities (if the independent variable is logarithmically transformed, e.g. income) [35]. The model is tested for sensitivity by including and excluding specific variables and by estimating the robust standard error. A series of diagnostic tests are performed, such as tests on the presence of heteroscedasticity, multicollinearity, and omitted variables.

Further, we predicted regression model using the WTP as proportion of income with the demographic and socioeconomic characteristics of the respondent. Since the dependant variable in this case is a proportion (WTP as share of income), the Generalized Linear Model (GLM) with binomial family and logit link function was applied as proposed by Papke & Wooldridge, 1996 [36].

## Results

### Characteristics of respondents

Out of the 557 respondents, 33.4% were rickshaw-pullers, 34.6% were shopkeepers and 32.0% were restaurant workers. The three occupation groups differed significantly in marital status, gender, age, education or household income.

However, the occupational groups did differ significantly in household size, educational level and household expenditure (Table 2). The correlation between monthly income and expenditure of the workers was 0.54 (p = 0.000).

**Table 2:** Respondent and household characteristics.

Variables	Rickshaw-puller	Shop-keeper	Restaurant worker	Difference across occupational group (p-value)	Total
Age	32.9	27.3	31.1	0.028	30.4
Gender (Male %)	99.5	98.5	87.6	0.000	95.3
Marital status (Married %)	82.8	37.8	64.6	0.012	61.4
Household size	4.6	5.5	4.8	0.072	5.0
Educational level					
Less than one year (%)	72	11	44	0.092	42
Up to primary (%)	23	33	36	0.073	30
More than primary (%)	5	56	20	0.051	28
Monthly income of the worker (BDT)	7,696.5	5,870.4	5,617.0	0.011	6,399.2
Household income per equivalent adult (BDT)	3,256.6	5,015.9	3,037.9	0.004	3,839.1
Household expenditure per equivalent adult (BDT)	2,948.7	3,473.6	2,328.3	0.998	2,965.2
Location					
Metropolitan city (%)	33.3	32.1	33.7		33.0
District (%)	34.4	36.2	34.2		35.0
Sub-district (%)	32.2	31.6	32.0		31.9
**Observations**	186	193	178		557

### Willingness to join in CBHI scheme and payment mode

86.7% of the respondents were willing to pay for CBHI. Respondents were offered two possible modes, weekly and monthly, for the payment of premiums; 63.4% chose the former and 36.7% chose the latter (Table 3).

**Table 3:** Distribution of participant WTP health insurance premiums weekly versus monthly by location and occupational group.

	Weekly payment	Monthly payment
**Locations**		
Sub-district	63.6%	36.4%
District	46.8%	53.3%
Metropolitan city	78.7%	21.3%
**Occupational groups**		
Rickshaw-puller	78.9%	21.1%
Shop-keeper	48.4%	51.6%
Restaurant workers	60.7%	39.4%
**Total**	63.4%	36.7%

Across the three locations, the metropolitan city location had the highest proportion of respondents (78.7%) who chose weekly payments, while the district town location has the highest proportion (53.3%) who chose monthly payments. The majority of rickshaw-pullers (78.9%) chose the weekly payment mode, while most shopkeepers (51.6%) chose the monthly payment mode.

### WTP for health insurance

The average WTP elicited by the bidding game was 22.8 BDT per week, with a 95% confidence interval (CI) of 20.9–24.8, which is approximately 20% higher than the median WTP (20.0 BDT). Average WTP was highest in the sub-district town location (27.0 BDT) followed by the metropolitan city (24.5 BDT) and the district town (16.6 BDT) locations. While the outliers were excluded, the average WTP was highest in Metropolitan city (22.5 BDT) followed by sub-district town (21.2 BDT) and district town (16.6 BDT). Average WTP was highest among rickshaw-pullers (28.2 BDT), followed by restaurant workers (20.4 BDT) and shopkeepers (19.2 BDT).

One-way ANOVA showed significant differences in WTP among locations and occupational groups (Table 4).

**Table 4:** WTP (mean and CI) per week across occupational groups and locations.

	Average WTP(BDT)(95% CI)	Average WTP excluding outliers (BDT)(95% CI)	Median WTP(BDT)	Significance test (p-value)
**Locations**				
Sub-district	27.0(22.5–31.6)	21.2 (18.9–23.4)	20.0	0.00
District	16.6(14.5–18.6)	16.6 (14.5–18.6)	12.5	
Metropolitan city	24.5(21.7–27.4)	22.5 (20.5–24.5)	20.0	
**Occupational groups**				
Rickshaw-puller	28.2(24.7–31.7)	25.0 (22.9–27.0)	20.0	0.00
Shop-keeper	19.2(16.1–22.4)	16.5 (14.4–18.6)	12.5	
Restaurant workers	20.4(17.0–23.8)	18.2 (16.2–20.2)	15.0	
**Total**	**22.8(20.9–24.8)**	**20.1 (18.9–21.3)**	**20.0**	**0.00**

### WTP and income

**Fig 1:**
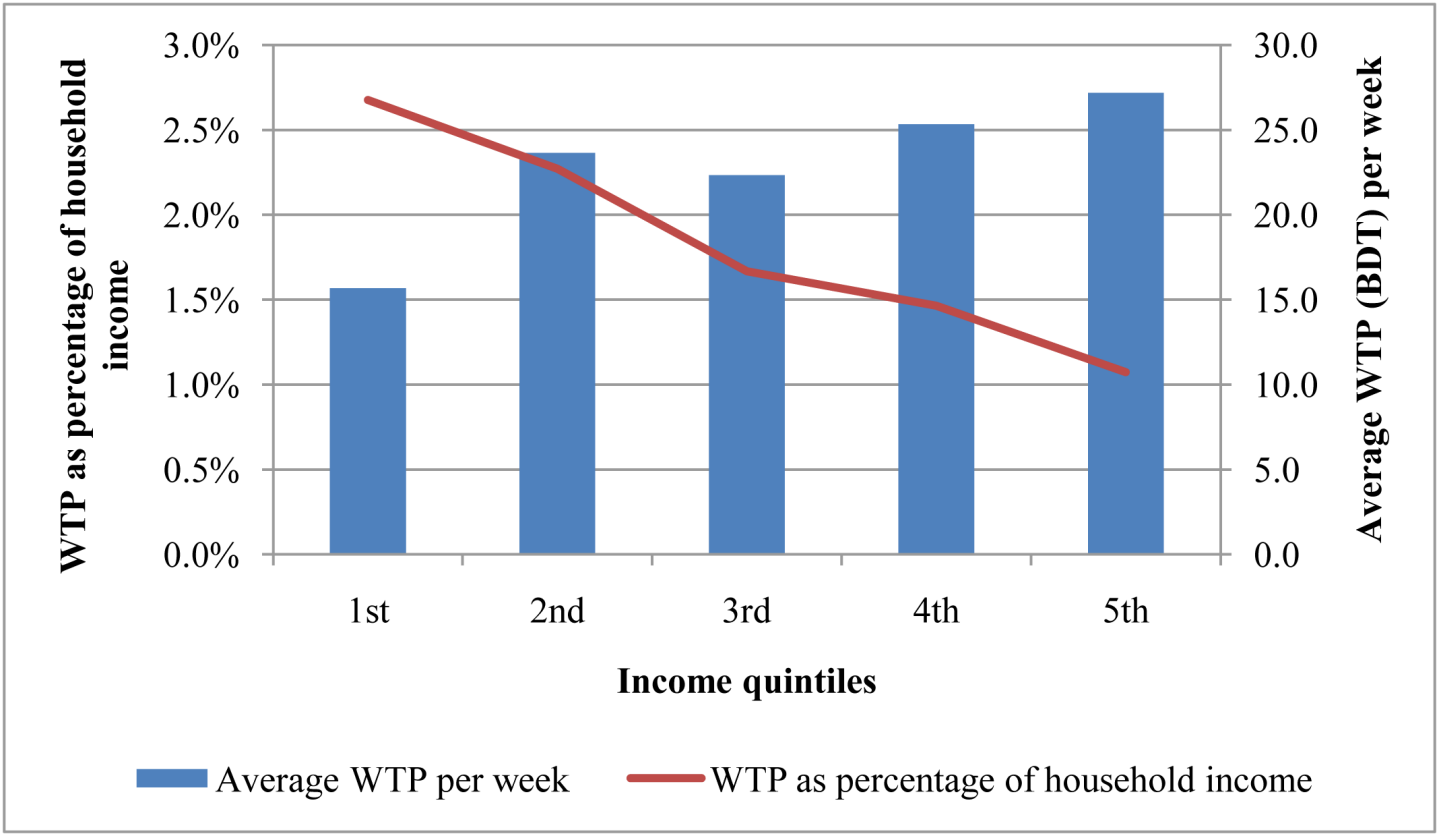
Average WTP and WTP as percentage of income (weekly) across income quintiles of the workers.

Fig 1 showed that the average WTP was higher among worker in richer income quintiles. However, the WTP as share of income was decreasing from lower income to higher income quintiles.

### Determinants of WTP

Regression analysis (Table 5) showed that educational level, monthly income, location and occupation significantly influenced WTP for CBHI among informal workers. Workers with up to a primary level of education were willing to pay 26.9% less than those with less than one year of education. Moreover, WTP increased a significant 0.196% with each 1% increase in monthly income of the worker. In the sub-district town and district town locations WTP was significantly lower (1.4% and 48.7% less, respectively) compared with the metropolitan city. WTP differed significantly among occupational groups. Shopkeepers and restaurant workers were willing to pay significantly less than rickshaw-pullers (68.5% and 38.6% less, respectively).

**Table 5:** Association of respondent characteristics with WTP (natural logged) for health insurance coverage from a multivariate regression analysis.

Variables	Description	Coefficient (Std. Err.)
Age	In years	-0.002(0.005)
Gender	Female (Ref = male)	-0.15(0.193)
Marital status	Unmarried (ref = married)	0.025(0.11)
	Others (ref = married)	0.29(0.749)
Household size	Number of household members	0.025(0.033)
Educational level	Up to primary level (ref = less than one year)	-0.269** (0.112)
	More than primary level (ref = less than one year)	-0.056(0.125)
Monthly income	Logged income per month	0.196** (0.077)
Illness in last 6 months	Illness of respondent or any household member	-0.01(0.125)
Location	Sub-district (ref = Metropolitan city)	-0.014*** (0.102)
	District (ref = Metropolitan city)	-0.487*** (0.105)
Occupation	Shop worker (ref = Rickshaw-puller)	-0.685*** (0.127)
	Restaurant workers (ref = Rickshaw-puller)	-0.386** (0.115)
Constant		1.83(0.672)
N		326
Adjusted R-square		0.219
F-value_(14,146)_ (Prob>F)		8.01 (0.000)
Mean VIF (max)		1.51 (2.24)
BP/Cook-Weisberg test (p>ch2)		0.45 (0.503)
Ramsey RESET, F (p>F)		3.46 (0.017)

Note

*** denotes significant at 1% risk level.

** denotes significant at 5% risk level.

The regression model explains 21.9% of total variations (R^2^ = 0.219). The Breusch-Pagan/Cook-Weisberg test showed that heteroscedasticity was absent from the model. The variance inflation factor (VIF) test obtained a maximum value of 2.24, which indicated no multicollinearity in the regression model. Ramsey RESET test showed sufficient evidence that the model did not suffer from omitted variable bias.

Robust standard error was calculated to test the robustness of the relationship between the magnitude of WTP (natural logged) and its determinants (Table 5). The regression model was reduced and extended by excluding and including variables. Determinants of WTP for the CBHI were similar for all models.

The GLM showed worker’s education level, monthly income, location and occupation were significantly associated with the WTP as share of income (Table 6). Households with higher income were more likely to have lower WTP as percentage of their income. Worker with primary education was less likely to have lower WTP as percentage than workers who have less than one year education.

**Table 6:** Association of respondent characteristics with proportion of WTP and income for health insurance coverage from a GLM regression analysis

Variables	Description	Odds ratio (95% CI)
Age	In years	0.99 (0.98–1.01)
Gender	Female (Ref = male)	0.83 (0.5–1.37)
Marital status	Unmarried (ref = married)	1.08 (0.83–1.39)
	Others (ref = married)	1.25 (0.65–2.42)
Household size	Number of household members	0.99 (0.96–1.04)
Educational level	Up to primary level (ref = less than one year)	0.66*** (0.52–0.85)
	More than primary level (ref = less than one year)	0.98 (0.71–1.37)
Monthly income	Logged income per month	0.46*** (0.34–0.61)
Illness in last 6 months	Illness of respondent or any household member	1.07 (0.71–1.61)
Location	Sub-district (ref = Metropolitan city)	1.25 (0.94–1.65)
	District (ref = Metropolitan city)	0.59*** (0.48–0.73)
Occupation	Shop worker (ref = Rickshaw-puller)	0.64** (0.45–0.9)
	Restaurant workers (ref = Rickshaw-puller)	0.75 (0.49–1.17)
Constant		25.25*** (2.28–279.84)
**N**	** **	**326**

Note

*** denotes significant at 1% risk level.

** denotes significant at 5% risk level.

WTP as percentage of income were lower among workers in district area than metropolitan city and among shop worker than Rickshaw-puller.

## Discussion

We found that a large proportion of informal workers (86.7%) were willing to pay for CBHI. They were willing to pay an average amount of 22.8 BDT (0.286 USD) weekly per household. This estimate varied across geographic locations and occupation of the respondents. Rickshaw-pullers were willing to pay the highest amount (28.2 BDT), followed by restaurant-workers (20.4 BDT) and shop-keepers (19.2 BDT). The largest WTP of rickshaw-pullers can be explained by their nature of income, e.g. they receive cash earnings every working day and need not to wait for weekly or monthly salary and their access to liquidity is higher and more frequent than other two occupation groups. WTP for health insurance varied across occupational groups, as did their preferred payment mode. The majority of rickshaw-pullers (78.9%) and Restaurant workers (60.7%) preferred weekly payment and shopkeepers (51.6%) chose the monthly payment.

A good number of literatures were published, which presented WTP for health insurance in low- and middle-income countries both in Asia and Africa. A study in Ghana found that almost 64% of respondents were willing to pay about Cedi 5000 (3 USD) per month per five-member household for a National Health Insurance scheme aimed at the informal sector [21]. Asgary et al. (2004) examined WTP for health insurance in rural Iran and found that households were willing to pay an average of 2.77 USD per month for health insurance [37]. In another recent study in Iran the average WTP for social health insurance per person per month was found 5.5 USD [38]. A study in Namibia found that uninsured individual in the Greater Windhoek Area of Namibia was willing to pay 47.50 NAD (6.60 USD) per month for individual health insurance [19]. Donfouet et al. 2011 found substantial demand for CBHI among rural households of Cameroon with willing to pay 1011 CFA francs (2.15 USD) per person per month [39]. An Indian study showed that median WTP was 55 INR (1.09 USD) per month [22]. Another study reported that people in rural India were willing to pay 1500 INR (27 USD) annually for CBHI [40]. Malaysia, a middle income country, using CVM approach revealed that more than 63.1% of the respondents were willing to join CBHI with an average payment of 114.38 USD per month per household [41]. In St. Vincent and the Grenadines, an upper-middle-income Caribbean country, 72.3% respondents were willing to join with WTP 77.83 EC$ (28.83 USD) per month per person to enroll in the National Health Insurance plan [42].

The multivariate analyses that we employed for predicting WTP for health insurance of informal workers suggested that the WTP was influenced significantly by educational level, monthly income, location and occupation of workers. Our finding of strong positive relationship between monthly income and WTP, was supported by other researchers [21,22,41,43,44]. Such findings can be justified by higher ability-to-pay of households with higher income level and WTP appeared to be higher with higher ability-to-pay [43].

While expected a positive relationship between educational level and WTP, we found, on the contrary, a significant negative influence of education. However, a previous study in urban China observed a similar outcome as ours [44]. In the context of Bangladesh, the knowledge about health insurance is not commonly available, especially among low-income people, given that only 0.1% of total health expenditure here was funded by private health insurance [5]. It was further observed that the geographic location of workers contributed to variations in WTP significantly. Asgary et al. 2004, Dror et al. 2007, Onwujekwe et al. 2010 and Binnendijk et al. 2013 also observed significant association between WTP and geographical locations [22,37,45,46]. Similar to this current study, several earlier studies observed significant association with occupation groups and WTP [19,42,47]. Respondent’s age showed no significant, but negative relationship with WTP. Several earlier studies found negative relationship between age and WTP [22,39,48]. The age variable may capture cohort effects. Age is plausibly not only proxies for disease risk but also associated with factors that are not controlled for in our regression analyses. For instance, older informal workers may believe that their children will finance their healthcare when they become ill, while younger workers do not [20]. A further model was tested using quadratic function of age (age×age) as an explanatory variable. It showed no significant relation with WTP. However the coefficient of age variable showed only 0.2% changes in WTP due to one unit changes in age.

The GLM showed WTP as percentage of income were more likely to decrease with increased income. This association follows the Engel’s laws which states that the proportion of income spent on food decreases as income increases, though the actual expenditure on food rises [49,50]. This finding indicated that health insurance is considered as a necessity good among informal worker. This was in line with the findings of Binnendijk et al. (2013), which showed that the rural poor in India considered health insurance as a necessity good with no prior experience of any insurance packages [46].

While most of our study findings were supported by other studies, there were some limitations. One potential limitation of CVM might be related to the respondent bias that might result from the starting bid [25,51]. To reduce the effect of such bias, we used various starting bid values ranging between 10 BDT and 30 BDT. Another important limitation of our study was that the interviews took place from December to April, and thus could not capture seasonal fluctuations in income of informal workers. However, usage of multivariate analysis in this paper considered workers with different income levels and might have captured proxy of seasonal variations in income to a good extent. The fundamental problem in estimating WTP in developing country context is that not many low-income people may understand the mechanism of health insurance scheme and the benefit package [31,52,53]. The poor understanding about health insurance mechanism can influence the demand or WTP for such product [54].

It was not very clear what portion of workers understood the insurance mechanism and benefit package to a good extent for making a rational choice on the amount they were willing to pay. It, however, can be argued that for minimizing the perception effect on decision making for WTP, we employed benefit packages that were available and familiar to workers in some areas (Public Health Centre in Dhaka and Savar) in Bangladesh. However, for establishing CBHI schemes there are other methods for developing benefit package of health insurance in developing countries through engaging low-income people [55,56].

Demand of health insurance, measured by WTP might not be influenced by the variables only that were applied in this study and in similar ones. Enrollment in health insurance had been affected by other variables according to some other studies. For instance, Roth et al. (2007) found that lack of knowledge about the importance of health insurance was an important determinant of health insurance product uptake [57]. From a client perspective, simplicity; affordability and value of micro health insurance products were found to influence the adoption of such product [31]. Even enrolment options and procedures [58] and proximity to quality healthcare facilities [59] had influence in joining health insurance. Therefore, the Cohen and Sebstad (2006) emphasized on the importance of creating awareness among the people on health insurance for creating demand [32]. It was even observed that people often do not trust the health insurance providers, which might have an impact on enrollment (Churchill, 2006). There were non-price frictions that could further limit demand; like limited trust and understanding of the product, product salience, and liquidity constraints. Rademarcher et al. (2010) argued that the trust to be developed in two dimensions; first the insurer is willing to make payments to clients, and second, the insurer is able to deliver the payments or services [60]. The improvements in insurance contract design and keeping the promise to beneficiaries can significantly mitigate these frictions [61]. It was, however, found in a study in India that a campaign using information and education had influence in health insurance enrollment [62].

CBHIs were criticized for poor designs with weak legislative, technical and regulatory frameworks which affect enrolment in CBHI and consequently the level of financial protection offered [63]. A systematic review of studies on impact of CBHI in low-income countries found strong evidence that it provides some financial protection and weak evidence that it affect quality of care [64]. These effects of CBHI are small in terms of population coverage [64]. However, CBHIs operating in countries (like, Ghana, Rwanda and Tanzania) with adequate legislative measures and government partnership was more successful in terms of coverage and risk protection [65,66]. Broader risk pools and proper design and implementation are important for mitigating the existing limitations of the CBHI schemes [67,68]. CBHI mechanism can play useful role where compulsory sources provide (like, enrollees) only minimal level of prepayment and redirect it to other prepayment pools (for instance, assistance from Government or international community) and CBHI can expand financial risk protection or healthcare seeking and help people to understand the benefits of being insured [11].

This current study provided the evidence on WTP for CBHI among urban informal sector workers and identified the major drivers of WTP. This finding will be useful in estimating the potential number of CBHI enrollees and potential revenue generation in low and middle income countries among the informal workers. Information on the prevailing preferences of the population for CBHI are limited in low- and middle-income countries. Policy makers, health care providers, and CBHI initiators thus can be benefited enormously from the estimates of WTP obtained in this study.

Universal Health Coverage, which is a global policy agenda as well as agenda for national governments in many low- and middle-income countries including Bangladesh, can find this study contributory when financial risk protection through risk pooling is considered [10,11]. The government of Bangladesh considered introducing CBHI scheme for informal workers in the healthcare financing strategy of the country [10]. Findings from this study could be useful for planning such schemes. In addition, other community-based models, like a public-community partnership model of healthcare financing in India (Rashtriya Swasthya Bima Yojana, RSBY) could be tried considering its experience, where government can be the guarantor and subsidize the premium [69].

## Supporting Information

S1 Dataset (DTA)
